# New York Medical and Surgical Society

**Published:** 1860-09

**Authors:** 


### SELECTIONS.

*New York Medical and Surgical Society.—Discussion on Diphtheria.*—Dr. ALLEN stated that since the last meeting he had met with another case of diphtheria, which he still had under treatment. A week ago, Thursday (June 26), a little German girl complained of a sore neck. These symptoms continued until the following Tuesday, when she experienced difficulty in swallowing. Her mother resorted to various domestic remedies, but to no purpose. The day following she was seized with a fever, which, along with the dysphagia, increased very much in severity, and I was sent for. I arrived in the afternoon about five o'clock, and found the patient very much prostrated ; the pulse 130 per minute, and weak ; breathing was rapid and somewhat labored, and there was considerable dysphagia present. I immediately had the child taken up, and on examining the throat, found both tonsils covered with the diphtheritic membrane, the uvula was slightly œdematous, and the pharynx was lined as far down as I could see. The examination was made with much difficulty, as it seemed to give the child a great deal of pain. I gave the citrate of quinine and iron, with brandy and beef tea internally, using locally Labarraque's solution. I saw the child the next morning, and found that the upper portion of the tonsil was less covered with exudation than it had been, but in other respects no change in the general symptoms was noticed. The treatment was continued, and at my visit in the afternoon I found still less of the exudation, and the pulse was more full and rapid. I then suspended the use of the citrate of iron and quinine, and ordered instead the spirits of mindererus with an excess of ammonia, continuing the beef tea and brandy. I also recognised that the case was complicated with pneumonia of the left side, which had passed into the second stage without the knowledge, on my part, of its existence before. This morning (Saturday), about four o'clock, I found the child suffering from croupy symptoms, and on inspection of the throat, I discovered that the exudation had almost entirely cleared off, only showing itself upon the epigolottis. I then stopped the sp. mind. and gave a grain of calomel every hour, and continued the rest of the treatment. At eight o'clock that morning I visited the case again, and at that time in company with Dr. Bloodgood. We decided to continue the administration of the calomel, and give quinine along with it. This afternoon about three o'clock I saw the child again, and found that the parents, thinking that the child was going to die, had discontinued the treatment about two hours before. There was very great difficulty in breathing, the air seemed to come through a perfectly dry tube, and at the end of two or three jerking sort of expirations, an inspiration would follow. I then told the father to give the child every fifteen minutes the syrup of ipecac until free emesis should follow. He did so. I called there just before I started to attend this meeting, and learned that in the interval three separate pieces of membrane had been expelled by vomiting, and that the patient in consequence felt very much easier. The last directions left with the father, were to repeat the administration of the remedy in the hope that some more of the exudation might be removed. The pneumonia has extended into the other lung, and there is now marked tubular breathing on both sides of the chest. Dr. A., in conclusion, mentioned that there was no scarlet fever in the family.

Dr. Bulkley stated that he had seen, within the last five years, four well marked cases of diphtheria, two of which had occured within the last two months. All these were connected, indirectly, with scarlet fever.

Dr. McCready stated that he had seen within two or three months two cases of diphtheria, which were by no means very severe, although in both instances they occured in very unfavorable subjects, they being of a marked strumous diathesis. The first child had a large patch of membrane on but one tonsil, the pulse was frequent, there was a good deal of soreness of the throat, and also some swelling about the glands of the neck. The severity of the disease was broken in a couple of days, the membrane commenced then to disappear, and at the end of the fifth day was gone entirely. The convalescnce was slow but perfect. In the second case the membrane existed on both tonsils, and the mode of its disappearance was the same as in the other case. The treatment in both consisted in the administration of the tr. mur. ferri in doses of three or four drops every two hours, together with wine whey, and good regimen.

Dr. Wilkes stated that he had seen three well marked cases of this disease, and all of them were quite severe, and quite characteristic. He did not think it was possible to confound it with croup or the ordinary sore throat.

Dr. Buck gave the following particulars of twelve cases of diphtheritic croup which he had met with since 1849. All these cases were children from three and a half to ten years of age ; seven were males, and five were females ; three were attacked in Oct., three in Nov., and one in each of the following months : Dec., Jan., Feb., March, April, and May. Of the twelve four recovered and eight died. Of the eight that died, six were tracheotomized; of the four that recovered, none were operated upon. All the twelve were unequivocal cases, and were complicated with laryngeal symptoms; with but two exceptions, the exudation was seen in the fauces, and upon one of these tracheotomy was performed. This patient, a young child, survived twenty-three days after the operation, and the wound, together with a blistered surface which existed before the operation, became covered with diphtheritic membrane.—In all, the early symptoms were those of sore throat, preceded in some instances by chilliness and fever, then followed by cough and the ordinary croupy symptoms. Those that were tracheotomized survived from thirty-six hours to twenty-three days, and all of them died in consequence of the extension of the disease into the air passages. In the child that survived the longest, there were convulsions complicating the case, and at the autopsy, there were discovered traces of the existence of pleuro-pneumonia. In all that recovered calomel was given, and the nitrate of silver was applied locally. In two of them the cinnibar fumigations were used in addition. In answer to a question from Dr. Bulkley, Dr. Buck stated that in all his cases the disease extended to the larynx and gave rise to croupy symptoms, and he was inclined to group them under the general head of diphtheritic croup.

Dr. McCready could not see where the dividing line could be drawn between diphtheria and croup. We discover a patch of yellowish membrane upon the tonsil, pharynx, and velum of a child who has a little sore throat, and is slightly feverish. The fever increases, the child begins to have difficult deglutition, and too soon the alarming symptoms of croup show themselves. That train of symptoms may last from three or four days to a fortnight, but how they can be distinguished from a case of diphtheria, I am at a loss to determine. They seemed to him to be the same disease.

Dr. Watson was of the opinion that diphtheria *was* croup, if the membrane extended into the larynx and gave rise to croupy symptoms.

Dr. Buck thought that the particular character of the disease depended upon a more or less inflammatory diathesis at different seasons of the year.

Dr. Clark stated that about one half his cases died in consequence of the effects of the disease upon the general system, and not of any mechanical obstruction. He had seen since the last meeting, three more cases, and of these one recovered. One was peculiar in certain respects, and bears upon the question whether or not this disease has any relation to scarlet fever. This patient was eight years of age. On the third of January the family physician was called, and on examination of the throat, discovered the existence of membranous matter upon the fauces, and he anticipated all the unpleasant results of its extension. The local treatment consisted in the application of nitrate of silver, together with five grains of quinine twice a day. In the course of four days the membrane became loosened and was removed by the forceps. At that time the disease showed no tendency to extend. Within twelve hours after, symptoms which finally ended in scarlet fever came on, and the throat during all that time was very sore. Recovery was commencing in the usual way, when the patient was seized with a new set of febrile symptoms, and in two days after, the full, irregular crescentic eruption of measles made its appearance. The measles took its usual course, and on the subsidence of the eruption new patches appeared on the fauces. On the third day after, this membrane reappeared, and I was called to see the case; then the fauces were covered; a broad patch of exudation which concealed the surface of the velum to a considerable extent, and also the tonsils, extended into the posterior nares, and forward so far that it could be seen in the nostrils. The physician had previously removed from the last-mentioned place, with a pair of curved forceps, long ribands of membrane. In a sort of vomiting effort, the child threw up a large quantity of tough leathery membrane. During all this time there was no marked obstruction to the respiration. When I saw the child, however, the respiration was exceedingly rapid, there was a moan at each inspiration, the pulse 140, and the surface and nails were blue. I predicted an unfavorable issue, notwithstanding there was no appearance of any membrane in the larynx. The evidence of poisoning of the blood became more and more apparent, the blue appearance of the surface continued, and in two days after she died. In the meantime the membrane had made its appearance upon a little sore on the lip, and had extended from it as a centre, a considerable distance over the surrounding apparently healthy tissue. The case is interesting when we take into account the fact that with this diphtheritic diathesis upon her, this girl went through scarlet fever and measles, had a very sore throat during the prevalence of the former disease, yet there was no diphtheritic membrane; and as the convalesence from measles was commencing the exudation appeared, and the disease progressed to a fatal termination without any serious obstruction to the air passages. In answer to a question from Dr. Elliot, Dr. Clark stated that he had met with no case that had terminated in convulsions.

Dr. Clark stated that on the evening after the last meeting he was called in consultation by Dr. Crane to visit a family in Elizabeth, N. J. Six, out of eight, children were suffering at the same time from scarlet fever, and one was lying dead in the house. Three out of the six children presented diphtheritic membrane in the fauces, and the remaining three had swollen tonsils with more or less inflammation of the throat. One of them had some white spots upon the inner surface of one of the tonsils, which at first looked a little like membrane, but afterwards turned out to be nothing more than a white secretion in the follicles. Two of them were at that time, as was supposed, desperately sick, and in one of these the membrane was distinctly discoverable in the nasal passages. The voice was a mere cry. The breathing was not so much obstructed as in croup, but sounded as if a valvular structure was playing up and down over the opening of the larynx; and we took it for granted that if the membrane had not already, it would eventually, extend into that portion of the breathing apparatus. The pulse was 140, and the intelligence nearly abolished. The patient was lying with her eyes closed, paying no attention to anything that was said, and considerable force had to be used to open the mouth. She moaned with almost every breath, though occasionally she would get a little quiet and seem to be asleep. This child finally recovered.

In one of the other children, the nasal passages were entirely plugged up by the drying of the secretions that flowed down from the external opening. The constitutional symptoms with him too were very marked. His pulse was the same as the others, but instead of being semi-comatose, he was restless, dozing continually. He lived nearly a week from the time I refer to, and apparently died from exhaustion, the result of the occurrence of numerous ulcerations very much after the manner of bed sores. It struck him that this latter feature of the disease was an evidence of the constitutional influence of the poison. The father, who was fifty-seven years of age, also had the diphtheritic exudation in the fauces, but in him none of the symptoms of scarlet fever had presented themselves. He, however, had the same character of valvular breathing as noticed in the daughter. His tonsils and velum were very much swollen, and the glands on the outside of the neck moderately so. The moment he lost consciousness in sleep, his breathing would stop as if something had passed into the opening of the larynx and prevented the entrance of air. The inspiration alone was obstructed. His friends were unwilling to allow him to sleep at such times for fear he would suffocate. This difficulty of breathing did not seem to me to be dependent upon the existence of a membrane, but upon the swollen condition of the hanging portion of the fauces, which dropped fairly down upon the top of the larynx. As soon as the inflammation subsided this symptom passed off. At the time we saw him he had been in a state of active delirium for forty hours; his pulse was about 100 per minute. He finally recovered. The treatment for all these cases was about the same; pretty active stimulation with alcohol and the very free use of sulphate of quinine, and the local applications of nitrate of silver in solution. There was a circumstance that interested me in connexion with the two children who had the membrane in its worst form, relative to scarlet fever. In the girl, the eruption was out full for eight days, and when we saw her was perhaps subsiding a little; in the boy, the symptoms had been out eleven days, and was still vivid. Desquamation was quite active, and the scales were standing out, attached to the surface by their edges, in all possible directions; rubbing these off, the eruption could be seen as on the second or third day. The urine in these cases was not examined.

Dr. Wilkes stated that he had met with an attack of diphtheria in a patient seventy-two years of age.

Dr. McCready within the last four weeks had been called to four cases of diphtheria following scarlet fever; two of these terminated fatally very soon after he saw them. In both the pulse was exceedingly frequent; there was a good deal of restlessness present, and the membrane covered the posterior part of the fauces, extending to the windpipe. The third case was somewhat similar in character as far as symptoms were concerned.

The first case was one of those which some time ago would have been called croup. I was called in consultation to see a stout boy, three years of age, with a pulse not much over 100, skin a little warm, and face somewhat flushed. I was told that there was ulceration about the throat, but no false membrane. On examination, however, the so-called ulceration was found covered with an ashy-colored patch of membrane, the child was also quite hoarse, and had the regular croupy cough. I did not see the case a second time, but read of its fatal termination a week after in the newspaper. The case agreed in every respect with the description which foreign writers give to croup.

Dr. Watts had seen one additional case since the last meeting:—A young lady, twenty years of age, was attacked no Wednesday last with what she supposed to be "chills and fever." She had a fair chill, followed by fever, a good deal of pain in the back, and also a sore throat. I was sent for on

Thursday afternoon, about thirty hours after she was first attacked. Her pulse was 130; she had a thickly-coated tongue, a severe pain in the back of the head and post-cervical region; the skin was cold and covered with a clammy perspiration. On looking into the throat, both tonsils were covered with a thick white deposit, which I am compelled to recognise as diphtheritic. I immediately placed her upon the use of quinine in two grain doses every two hours, and directed wine-whey to be given with the utmost freedom. Hér skin was rubbed to get up an active circulation, and at bed-time opium was added to the quinine. I saw her yesterday morning, and the symptoms were decidedly moderated. Yesterday she was a good deal better; and to-day I found her very comfortable. The exudation has disappeared, leaving in its place a strawberry-roughness. The pulse is about 90, and has considerable force. No local treatment was employed.

Dr. METCALFE next made the following statement:—Since the beginning of the winter I have had ten cases of this disease, six of which I have seen in consultation. There have been seven cases in which the diphtheritic deposit affected the throat mainly, in the others the Schneiderian membrane was the principal seat of the exudation. The first case was a child three years of age, who was dying when I saw it; both tonsils and a part of the velum were covered with the membrane. The patient died comatose. The next was the sister of this child, who presented the exudation on each tonsil, the palate, and in the nostrils; there was a good deal of constitutional excitement, with occasional delirium, present. This case terminated favorably after a fortnight's illness. The third case was a brother of the last, eight months old; the membrane was situated on the surface of the tonsils, and invaded a small extent of the palate. This child recovered after four weeks illness. The uncle, who was in the house, convalescent from measles, had a slight diphtheritic patch on the palate. The mother also, had some trouble about the throat, her tonsils were much reddened, and the peculiar coating could be scraped from their surfaces without much difficulty. The constitutional disturbances were very trifling, and in two or three days she was entirely recovered. The next was a little girl four years old; I saw her on the next morning after the night she was attacked, when I found both tonsils almost completely covered with the membrane. The pulse ranged from 160 to 180. The breath was horribly fetid. The exudation in the course ot the next day spread so as to cover the palate, and the grave symptoms increasing, the child died of apnœa two days after. The next was a child twelve years old, of a delicate constitution, who was taken on a Sunday morning, the membrane covering both tonsils and the edge of the soft palate. On Monday she was somewhat better; on Tuesday the fever subsided, and the membrane disappeared. That night the membrane reappeared, and extended into the nostrils; together with this there was attendant an immense tumefaction of one side of the neck. In consequence of this, there was a good deal of constitutional excitement, delirium, and difficulty of deglutition. The child, after making us believe for the greater part of four days that she was going to die, finally became convalescent. In this connexion Dr. Metcalfe exhibited a beautiful cast of membrane which had separated itself from the tonsils. Another case was of a young man, a member of the class at the University. He was taken sick on Saturday, and showed the patches in his throat the day following, when he experienced some difficulty in deglutition; had fever debility and quickness of the pulse. These symptoms continued for three days; he suffering a great deal without being, as I thought in positive danger. On the fifth day after the commencement of the attack, he was suddenly taken with a rigor, his skin was cold and covered with perspiration—respiration forty per minute. He could not lie down for a minute without having symptoms of suffocation. The gentleman who saw him with me was of the opinion that the case would terminate fatally very soon; the patient, however, recovered, and was able to return home on the Monday following. These are the only cases worthy of mention; of the rest, with but one exception there was very little constitutional excitement—some quickness of the pulse, pallor of the body, restlessness, pain in swallowing, and the occurrence of a well-marked membrane, with nasal defluction—and they all got well. I have not used quinine in any of these cases, but in its stead the mur. tinct. ferri in twenty-drop doses every two hours to adults, decreasing the quantity according to the age of the patient. Besides this, I give plenty of beef-tea, milk-punch, and wine whey. I have used the sol. of nit. silver locally, but can't say that I have derived any benefit from it. I have given the chlorate of potash as a gargle, but there again I failed in obtaining any good results. In conclusion, Dr. Metcalfe referred to a new remedy, the iod. of bromine, which had been brought to his notice by a physician in Long Island. It was used locally in the strength of fifteen drops to eight ounces of syrup, and was of great service in correcting the fetor of the breath. He (Dr. M.) had succeeded very well with the remedy, and advised the members to give it a trial.

Dr. Watson referred to the case of a gentleman who, within the last six weeks, was attacked four different times with sudden fits of suffocation, which, after existing for a time, would be followed by the discharge of a plug from the bronchial tubes, when immediate relief would ensue. Dr. W. attended him in one of these attacks, and stated that the plug raised at that time was about two inches long, and about as thick around as the forefinger. The extremities of this mass were much softened while the centre was hard and tough. He thought it possible that the condition of things referred to might have more or less to do with the epidemic of diphtherite.—*Am. Med. Times.*

